# Self-objectification and eating disorders: the psychopathological and neural processes from psychological distortion to psychosomatic illness

**DOI:** 10.1093/psyrad/kkae003

**Published:** 2024-02-28

**Authors:** Yinying Hu, Yafeng Pan, Liming Yue, Xiangping Gao

**Affiliations:** Department of Applied Psychology, School of Psychology, Shanghai Normal University, Shanghai, Shanghai 200234, China; Department of Psychology and Behavioral Sciences, Zhejiang University, Hangzhou, Zhejiang 310058, China; The State Key Lab of Brain-Machine Intelligence, Zhejiang University, Hangzhou, Zhejaing 310058, China; Department of Applied Psychology, School of Psychology, Shanghai Normal University, Shanghai, Shanghai 200234, China; Department of Applied Psychology, School of Psychology, Shanghai Normal University, Shanghai, Shanghai 200234, China; Shanghai Institute of Early Childhood Education, Shanghai Normal University, Shanghai, Shanghai 200234, China

Eating disorders are a kind of serious psychosomatic illness characterized by abnormal eating habits and thoughts regarding food and body weight (Treasure *et al*., 2020). They are highly prevalent worldwide, particularly among adolescent girls and young adult women (Arcelus *et al*., 2011). For example, >9% of the US population suffers from eating disorders, and 22.2–31.6% of children and adolescents are at risk of developing eating disorders (He *et al*., 2017; Sparti *et al*., 2019). Around 5% of patients also experience symptoms of the illness throughout their entire lifetime, enduring long-term malnutrition and severe complications (Treasure *et al*., 2015), and >10% of patients experience suicidal ideation or behavior (Conti *et al*., 2017). Thus, the identification of individuals at greater risk for developing eating disorders and the proper implementation of early prevention and treatment strategies hold significant importance.

Eating disorders can arise due to a variety of factors, including the societal emphasis on physical appearance (Ata *et al*., 2015). When an individual places excessive importance on their physical appearance, it is referred to as self-objectification (Fredrickson & Roberts, 1997). Self-objectification is a psychological issue, consistently accompanied by habitually monitoring and being dissatisfied with one's body (Ward *et al*., 2023; Zheng *et al*., 2015). It emphasizes physical appearance over other attributes such as abilities, personality, and temperament, and includes evaluating one's own appearance from an external perspective, overlooking personal feelings and needs. Crucially, self-objectification has been linked to the presence of eating disorders, as demonstrated by correlational studies indicating that increased objectification of one's body is associated with elevated levels of disordered eating attitudes and behaviors (Calogero, 2009; Dakanalis *et al*., 2015; Daubenmier, 2005; Geng, 2020; Guo *et al*., 2021; Han *et al*., 2019; Jongenelis *et al*., 2014; Kilpela *et al*., 2019; Lindner *et al*., 2012; Moradi & Huang, 2008; Niu *et al*., 2020; Rodgers & Melioli, 2016; Tiggemann & Williams, 2012; Yao *et al*., 2018; Zhao & Jiang, 2021; Zhang & Zeng, 2023). In one study that observed a correlation between self-objectification and eating disorders (*r* = 0.39), a stronger effect was presented in women (*r* = 0.41) than in men (*r* = 0.20; Schaefer & Thompson, 2018). The link was also more pronounced in white (*r* = 0.42) and Asian American (*r* = 0.42) women compared to African American or Black (*r* = 0.34) women, and it was most robust among heterosexual women (*r* = 0.39) while being weakest among heterosexual men (*r* = 0.23; Nechita *et al*., 2021).

A significant proportion of individuals who experience self-objectification may develop eating disorders, but there is still no consensus on the mechanisms underlying this relationship. In this review, we provide a summary of psychological and neuroimaging research on the relationship between self-objectification and eating disorders, focusing on studies that have investigated the mechanisms involved in this link. First, we examine the psychopathological pathway of self-objectification. Second, we discuss the pathological mechanisms that connect self-objectification to eating disorders. Third, we discuss the neural mechanisms that underlie the connection. Finally, we propose directions for future research.

## The psychopathological pathway of self-objectification

Self-objectification is a consequence of sexual objectification, which treats women as mere bodies or collections of body parts (Bernard *et al*., 2020). Generally, women are exposed to sexual objectification stimuli (i.e. bare body, sexy pose, and appearance evaluation) almost every day via television programs, music videos, movies, WeChat Moments, Weibo Square, and other social media, as well as daily interpersonal interactions. For example, it was revealed on analyzing 662 television programs that 96% of female actors were shown with their bodies exposed, but only 68% of male actors were shown wearing revealing clothing (Flynn *et al*., 2015). In another instance, a multinational sample of women aged 18–46 years reported experiencing an average of 2.7 incidents of interpersonal sexual objectification over a 5-day period (Koval *et al*., 2019).

Following self-objectification, a series of psychologically pathological risks often ensue, particularly in relation to eating disorders. Specifically, self-objectification signals an imbalance in self-concept, leading to negative outcomes such as reduced internal sensitivity, increased negative emotions, and impaired self-regulation (Ward *et al*., 2023). There is empirical evidence that individuals with self-objectification place an excessive emphasis on their appearance, considering it much more important than their competence (Vandenbosch *et al*., 2015). In addition, it has been shown that greater self-objectification is associated with less sensitivity to internal states (Ainley & Tsakiris, 2013), more negative emotions (such as shame, low self-esteem, anxiety; Dvir *et al*., 2021), and decreased self-regulation (Baker *et al*., 2017). For instance, individuals with greater self-objectification tended to feel less cold when wearing limited clothing outside a nightclub on a cold night (Felig *et al*., 2022), and they also performed more poorly on the Stroop test (Ching & Wu, 2022). A large number of studies have established a correlation between self-objectification and psychological issues, including depression and anxiety (Lindner & Tantleff-Dunn, 2017; Sun, 2021; Teng *et al*., 2019).

Self-objectification is essentially a form of rumination on the state of one's own body. That is, repetitive, prolonged, and recurrent negative thinking about one's own body, appearances, personal concerns and upsetting experiences. Earlier research has suggested that self-objectification may have more pronounced effects in individuals who are more inclined to ruminate in response to negative thoughts (Calogero & Jost, 2011). Furthermore, there is evidence that individuals experiencing self-objectification may become ensnared in ruminative thoughts about their own bodies, resulting in a decline in their overall sense of well-being (Jarrar, 2017). Taken together, self-objectification encompasses the concurrent presence of both body and emotion, persistent negative thought patterns, and the reciprocal reinforcement of negative emotions. These factors culminate in chronic depletion and regulatory dysfunction due to insufficient resources, ultimately elevating the risk of developing disordered eating behaviors.

## The psychopathological mechanisms linking self-objectification and eating disorders

Early theory and research suggested that the link between self-objectification and eating disorders was mediated by body shame (Calogero, 2009; Fredrickson & Roberts, 1997). However, a longitudinal model did not confirm this explanatory pathway (Kilpela *e t al*., 2019). Another hypothesis, known as the allocentric lock hypothesis, suggests that the progression from self-objectification to eating disorders is related to the inability to update one's internalized body image with current sensory input through self-integration (Riva *et al*., 2015). Evidence has indicated that both individuals who self-objectifiers objectify and those with eating disorders show lower memory performance compared to the controls (Pacilli *et al*., 2016; Serino *et al*., 2015).

Here, we further suggest that self-rumination may bring out the relationship between self-objectification and eating disorders. Eating disorders are the result of self-criticism that is reinforced by rumination on negative body perceptions. Patients with eating disorders have a persistent negative body image, which extends to a negative scheme for self-concept (Riva & Dakanalis, 2018). A meta-analysis of 38 studies revealed that rumination was significantly associated with eating disorders both concurrently (*r* = 0.33) and prospectively (*r* = 0.23) and also that individuals with eating disorders exhibited greater levels of rumination compared to control participants (Smith *et al*., 2018). Moreover, in experimental studies that induced feelings of sadness or concerns related to eating or body image, and subsequently manipulated state rumination, it was noted that in women with eating disorders, rumination led to heightened negative mood, diminished body satisfaction, and increased analogue symptoms (e.g. the desire to binge or abstain) compared to conditions involving acceptance or distraction (Naumann *et al*., 2015, 2016).

Notably, the rumination observed in those with eating disorders is not limited to the psychological level (negative self-schemas); it is also present as behavioral rumination. This dual presence of rumination (psychological processes and behaviors) indicates that eating disorders are a disease that involves an interaction between the mind and the body. Thus, single physiological or psychological interventions have limited effectiveness in the treatment of eating disorders; as one example, medication, one of the most commonly used treatments, has shown limited effectiveness in addressing the psychological, somatic, or nutritional aspects of eating disorders (Blanchet *et al*., 2019). Despite receiving the most effective treatment (cognitive behavioral therapy), >60% of patients continue to display the core symptoms of eating disorders (Slade *et al*., 2018). On average, the effectiveness of current intervention methods is moderate, with remission rates ranging from 40 to 60% in cases of anorexia nervosa, bulimia nervosa, and binge-eating disorder (Eddy *et al*., 2017; Linardon, 2018).

## The neural mechanisms linking self-objectification and eating disorders

The brain activity in the self-referential network may indicate a transition from self-objectification to eating disorders. Previous neuroimaging research has demonstrated the role of the self-referential network in self-objectification (Cikara *et al*., 2011; Cogoni *et al*., 2018; Du *et al*., 2023), self-rumination (Int–Veen *et al*., 2023; Laicher *et al*., 2022; Rosenbaum *et al*., 2021), and eating disorders (Brooks *et al*., 2011; Martins *et al*., 2020; Spangler *et al*., 2012). Specifically, individuals with higher levels of self-objectification show decreased brain activity in the self-referential network, including the medial frontal cortex, inferior frontal cortex, inferior parietal cortex, superior temporal cortex, and cingulate gyrus (Sun *et al*., 2016). However, it exhibits a distinct pattern during ruminative processes, as well as in individuals with eating disorders. One meta-analysis, involving experimental tasks examining rumination from 14 fMRI studies with a total of 286 healthy participants, has confirmed the link between self-rumination and activation of the self-referential network (Zhou *et al*., 2020). This association is specifically attributed to the dorsal medial prefrontal cortex subsystem, which is believed to promote self-generated thoughts, which are based on internally constructed representations rather than external ones (Buckner & DiNicola, 2019). In eating disorders, individuals demonstrate increased activation in the self-referential network compared to healthy controls when viewing food images (Brooks *et al*., 2011), performing cognitive control tasks (Seitz *et al*., 2016), and evaluating body and self-images (Spangler *et al*., 2012). This activity of the self-referential network could potentially signify the presence of negative thoughts and emotions concerning body image, such as ruminative preoccupation with body shape and anxiety about gaining weight.

The connectivity within the self-referential network might also contribute to the progression from self-objectification to eating disorders. Within the self-referential network, a core subsystem is present, comprising the medial prefrontal cortex, the posterior cingulate cortex, and the inferior parietal lobules. This subsystem serves as the foundation for self-relevant cognition, demonstrating consistent engagement across mental states involving self-oriented processing (Delahoy *et al*., 2022). When individuals engaged in rumination, there were increased connections between the core and the medial temporal lobe subsystem, while decreased connections between the core and the dorsal medial prefrontal cortex subsystem (Chen *et al*., 2020). Enhanced functional connectivity might signify immersion of oneself in the past, surrounded by negative thoughts, while decreased functional connectivity could be interpreted as a reduced focus on the present. The altered connections within the self-referential network are often associated with symptoms of eating disorders and other psychological illnesses (Chen *et al*., 2021). For instance, depression patients exhibited a more negative connectivity from the medial prefrontal cortex to the posterior cingulate cortex compared to healthy individuals, indicating an excessive regulation of the posterior cingulate cortex by the medial prefrontal cortex (Davey *et al*., 2017). In another example, participants with social anxiety disorder exhibited heightened excitatory connectivity from the posterior cingulate cortex to the medial prefrontal cortex and increased inhibitory connectivity from the inferior parietal lobule to the medial prefrontal cortex. These findings reflect the characteristic fear of negative social evaluation experienced by individuals with social anxiety disorder (Jamieson *et al*., 2023).

Besides the self-referential network, the connectivity between the self-referential, cognitive control, and body perception networks plays a role in the progression from self-objectification to eating disorders. For instance, the stronger the connection between the self-referential network and the somatosensory cortex, particularly in the secondary somatosensory cortex and ventral premotor cortex, the greater the level of self-objectification (Du *et al*., 2023). These connections indicate a heightened focus on external attributes of self-related sensory information in self-objectifiers, such as physical appearance. In eating disorders, the changes in connectivity between the self-referential, cognitive control, and body perception networks are found to be linked with more frequent eating-disorder behaviors (Chen *et al*., 2021; Domakonda *et al*., 2019; Frank *et al*., 2021; Gaudio *et al*., 2016; Haynos *et al*., 2021). In one recent study, high body image concerns and bulimic behaviors were correlated with increased connections within the cognitive control network, as well as between the cognitive control network, self-referential network, and visual perception network, with decreased connections present between the reward sensitivity network and visual perception network (Chen *et al*., 2023). This altered neural connectivity suggests the existence of some potential abnormal pathways underlying eating disorders, such as aberrant integration between information perception and reward valuation, and excessive rumination on self-referential thoughts about body image. Altogether, the activity and connectivity within and between the self-referential, cognitive control, and body perception networks could suggest a shift from self-objectification to the development of eating disorders.

## Challenges and future directions

According to objectification theory (Fredrickson & Roberts, 1997), the involvement of self-objectification in the development of eating disorders is strongly implicated, and numerous studies have been conducted to empirically investigate the relationship between these constructs. Still, there remain outstanding challenges to examining the pathological pathway leading from self-objectification to the development of eating disorders, especially the underlying neural mechanisms.

First, scholars should broaden their research on the pathological characteristics and development of self-objectification by conducting longitudinal studies, as the use of cross-sectional data in previous studies has limited their ability to definitively identify the mechanism of self-objectification (Schaefer & Thompson, 2018). This may involve collecting online surveys that evaluate self-objectification, body surveillance, body shame, body dissatisfaction, and eating disorder pathology at the initial assessment, as well as 6-, 12-, and 24-month follow-ups. Second, since the self-referential, cognitive control, and body perception networks are now known to play a crucial role, research approaches must evolve accordingly, especially within investigations aimed at examining the association between the changes in these networks and the pathological characteristics of self-objectification, as well as the causal relationship with the development into eating disorders. Future studies could explore the particular neural networks that predict self-objectification characteristics, such as body surveillance, body shame, and body dissatisfaction, within the discovery sample of individuals. Additionally, they might investigate the specific neural networks that predict symptomatology associated with eating disorders, such as body image concerns, binge eating, and compensatory behaviors, within a cohort of self-objectifiers. Third, future research should investigate the feasibility of using neurobiological alterations in the self-referential network as diagnostic indicators for self-objectification, as well as a predictive indicator for the progression from self-objectification to eating disorders. Indeed, recent neuroimaging studies have attempted to identify predictors of risk factors across eating disorders, which offers a new direction for research (Chen *et al*., 2023). Future studies could use objectified material to initiate a state of self-objectification and to measure the activity of the self-referential network before and after priming. Fourth, it would be beneficial to use the brain activity and connectivity in the self-referential, cognitive control, and body perception networks as diagnostic and evaluative indicators for understanding the pathology and intervention effects of eating disorders. Future research could involve tracking a cohort of individuals experiencing self-objectification and analyzing both their brain activity and connectivity before and after the onset of eating disorder symptoms. Finally, recent studies have described the important contributions of non-invasive brain stimulation for the management of eating disorders (Duriez *et al*., 2020). By using techniques such as magnetic or electrical stimulation, future studies have the potential to modify the pattern of brain activity and connectivity associated with eating disorders, while measuring eating disorder symptoms before and after the stimulation.

In conclusion, empirical neuroimaging studies are needed in the future to clarify how self-objectification contributes to the development and maintenance of eating disorders. This knowledge could help inform prevention and intervention strategies and guide the development of targeted treatments.
